# Evaluation of the Antiviral Activity of Monomeric, Dimeric, and Oligomeric Flavonoids against Chikungunya and Mayaro Viruses

**DOI:** 10.1021/acsomega.6c02683

**Published:** 2026-07-16

**Authors:** Delaine Meireles Gouvêa, Maria Cecília Muniz de Souza Brand, Millena Alves Máximo Vaz, Priscila Gonçalves Ferreira, Vinicius Viana, Kamilly Soares de Almeida, Ariane Coelho Ferraz, Adriana Cotta Cardoso Reis, Geraldo Célio Brandão, Cintia Lopes de Brito Magalhães, José Carlos de Magalhães

**Affiliations:** † Departamento de Química, Biotecnologia e Engenharia de Bioprocessos, Universidade Federal de São João del-Rei Campus Alto Paraopeba, Rod. MG 443, KM 7 Fazenda do Cadete, CEP: 36495-000 Ouro Branco, Minas Gerais, Brazil; ‡ Programa de Pós-Graduação em Ciências Biológicas, Núcleo de Pesquisas em Ciências Biológicas, 28115 Campus Universitário, Morro do Cruzeiro, s/n, Vila Itacolomi, CEP: 35400-000 Ouro Preto, Minas Gerais, Brazil; § Programa de Pós-Graduação em Ciências Farmacêuticas, Escola de Farmácia, Campus Universitário, Morro do Cruzeiro, s/n, Vila Itacolomi, CEP: 35400-000 Ouro Preto, Minas Gerais, Brazil

## Abstract

This study evaluated a panel of structurally diverse flavonoids, including monomeric, dimeric, and oligomeric compounds, to investigate the influence of structural complexity on antiviral activity against Chikungunya (CHIKV) and Mayaro (MAYV) viruses. CHIKV and MAYV, which are both members of the genus *Alphavirus*, represent a growing public health concern in Brazil. This is owing to rising arboviral infections and the potential for cotransmission by shared urban vectors. Effective control requires integrated strategies, including antivirals. Therefore, this study evaluated the cytotoxicity, antiviral activity, and *in silico* pharmacokinetic/toxicological profiles of nine structurally diverse compounds. Accordingly, procyanidins A2 (PA2), C1 (PC1), and genistein (GNT) displayed the most promising antiviral activity against CHIKV. Moreover, these compounds exhibited selectivity index (SI) values of 5.9, 10.4, and 2.6, with half maximal effective concentrations of 56.7, 50.2, and 64.4 μM, respectively. Furthermore, for the anti-MAYV activity, PA2 and PC1 exhibited SI values of 4.1 and 61, with EC_50_ values of 81.7 and 8.8 μM, respectively. In addition, GNT induced a 5-log reduction of CHIKV replication, whereas PC1 caused a 4-log reduction of MAYV replication. In virucidal assays, PA2 and PC1 nearly abolished (∼100%) CHIKV infectivity and reduced MAYV infectivity by approximately 80%.*In silico* analysis indicated that GNT had favorable pharmacokinetics and high toxicological safety, whereas PC1 demonstrated strong safety despite limited oral bioavailability. These results identify PC1 as the most promising candidate for future preclinical development, whereas GNT and PA2 should be considered lead compounds for further optimization strategies aimed at improving selectivity and safety profiles.

## Graphical abstract

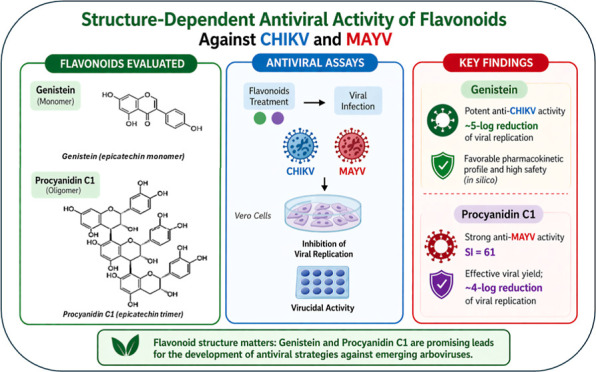

## Introduction

Viral infections account for a large proportion of clinical reports, particularly in the tropical regions of Latin America, where diseases, such as Chikungunya, Zika, and Dengue fever, are prominent. Additionally, diseases with similar symptomatology, such as Mayaro and West Nile fever, remain neglected. Consequently, some of these diseases and case numbers occasionally increase, and outbreaks progress into epidemics, causing further harm.^1,2^

Brazil is a tropical country; therefore, the climate, vegetation, and biodiversity increase the incidence of arboviral diseases, which are caused by arthropod-borne viruses. Simultaneously, their potential transmission in endemic urban areas by viral vectors, such as *Aedes aegypti*, poses an additional risk. The widespread circulation of arboviruses, such as Chikungunya virus (CHIKV) and Mayaro virus (MAYV), poses a threat to the country’s public health.^3–5^

The species *Alphavirus chikungunya* and *Alphavirus mayaro* are enveloped viruses with a single-stranded, positive-sense RNA genome that belong to the genus *Alphavirus* and family *Togaviridae*. Hence, these viruses share a close phylogenetic and structural relationship.

Infections by these viruses induce symptoms, such as fever, headache, and chronic arthralgia, in 72–95% of patients. In the case of MAYV, acute meningoencephalitis may occur. Some patients report persistent joint pain that lasts for years, leading to movement limitations, inability to work, depression, and even death.^6–9^

According to the Brazilian Ministry of Health, in Brazil (2025) 129,123 probable cases of chikungunya were reported, including 121 deaths, of which 107,975 were confirmed. In 2026, 4544 probable cases were reported, of which 1535 were confirmed, with no deaths recorded. In terms of MAYV, these data are underreported as this virus is often mistaken for CHIKV or Dengue virus (DENV) owing to their similar symptoms.^10^

In 2023, the Food and Drug Administration, in partnership with the Butantan Institute in Brazil, approved the production of a vaccine effective against CHIKV, thus marking a milestone in the global fight against the disease. In 2025, the vaccine was approved in Brazil by the National Health Surveillance Agency and vaccination has already begun in pilot cities, and it will be prioritized in endemic areas, where there is a higher incidence of the disease. The expectation is that the vaccine will soon be incorporated into the Unified Health System (SUS), expanding vaccination coverage in country.^11^ However, effective control of pathogens with this profile requires three pillars: vector control, vaccine use, and the availability of antivirals. In this regard, different bioactive molecules may be candidates for antiviral agents, including flavonoids, which are a prominent class of plant-derived molecules that have been widely studied for different biological functions.^12,13^ Their biological effects include anti-inflammatory,^14^ antidiabetic,^15^ anticancer,^16^ neuroprotective,^17^ antioxidant,^18^ and cardioprotective.^19^ In terms of antiviral activity, flavonoids act at different stages of infection, potentially blocking the entry of viruses into cells and interfering with viral genome replication or hindering viral release at the end of the cycle. In addition, flavonoids stimulate the immune response, thereby aiding in the defense against and elimination of viruses.^13,20^

Antiviral compound discovery and elucidation of their mechanisms of action, with a particular focus on arboviruses, has become a popular research topic.

Broad-spectrum antiviral activity has been described for certain groups of flavonoids. The set of nine flavonoids was selected based on a criterion of structural diversity to explore the influence of the degree of polymerization (monomeric, dimeric, and oligomeric) and the chemical skeleton on the antiviral activity against CHIKV and MAYV. The selection included monomeric compounds (such as isoflavones, flavonols, and flavan-3-ols) that represent distinct classes of flavonoids. Additionally, dimeric compounds (such as type A and B procyanidins, and the biflavonoid amentoflavone) and an oligomeric compound (procyanidin C1) were included, all known to exhibit biological properties distinct from monomeric structures. The primary goal of this structurally diversified approach was to identify whether the increase in molecular complexity and size correlates with higher antiviral potency or selectivity, providing a crucial comparative basis for the development of new drug candidates against the studied arboviruses (Figure 1). There are no studies that clearly link whether different structures can alter the antiviral action of molecules.

**Figure 1:**
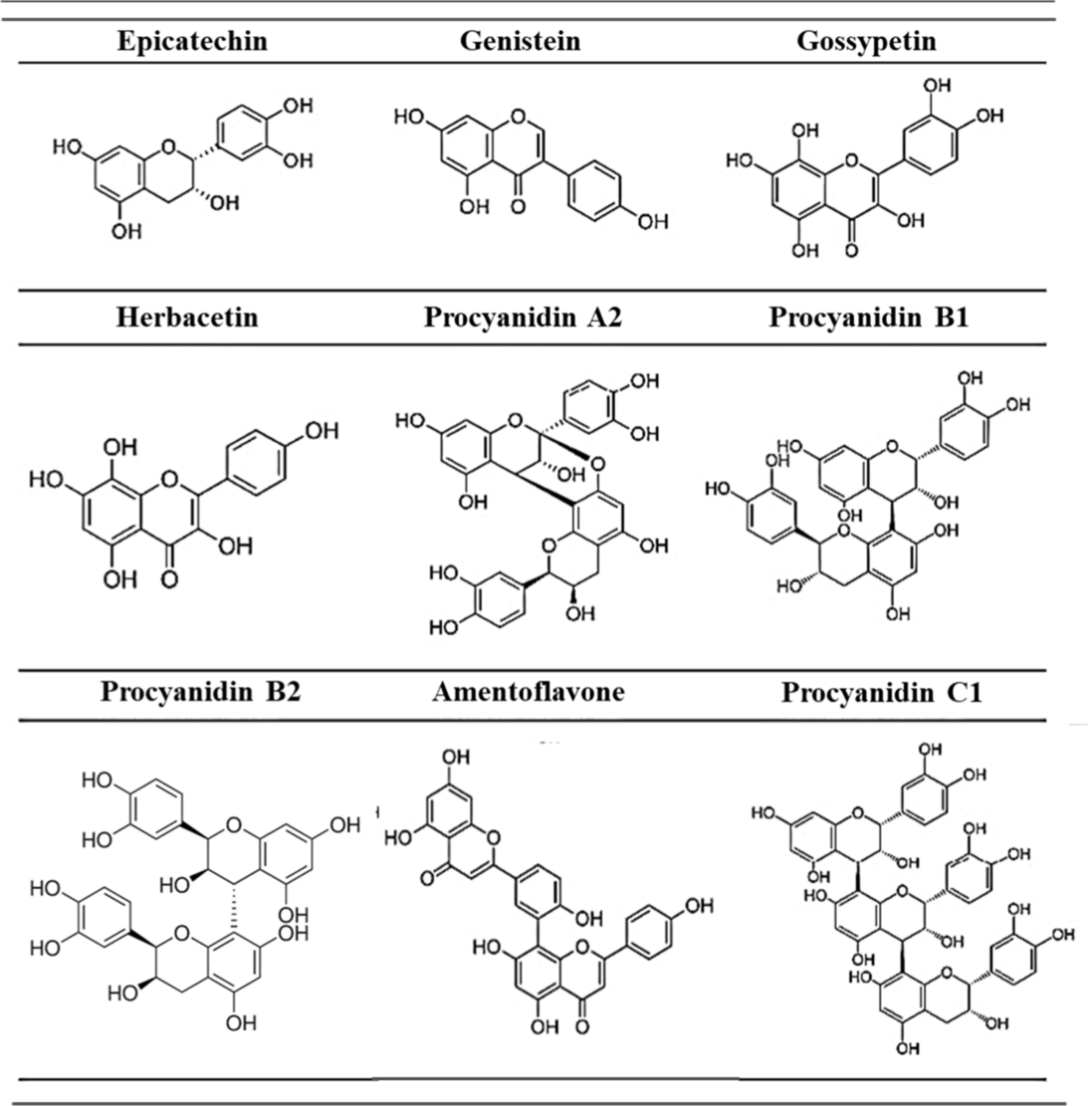
Chemical structures of the evaluated flavonoids. Monomeric compounds: Epicatechin, Genistein, Gossypetin, and Herbacetin; dimeric compounds: Procyanidins A2, B1, and B2, and Amentoflavone; oligomeric compound: Procyanidin C1.

## Results

### Effect of Selected Compounds on Vero Cell Viability

The MTT assay was performed to determine the CC_50_ of the nine flavonoids. Cell viability results are shown in Figure 2 and were used to identify the most promising compounds.

**Figure 2:**
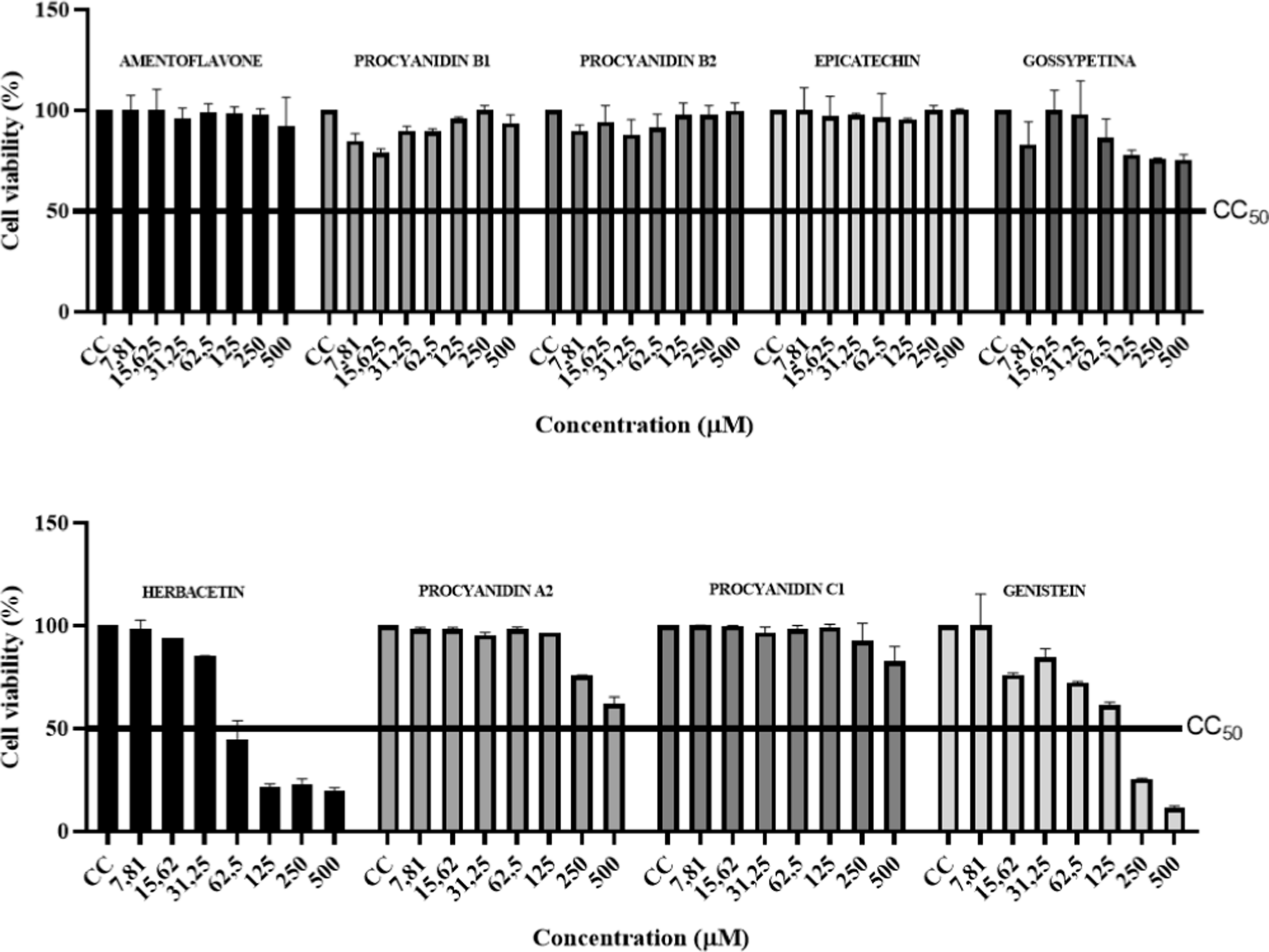
Cell viability after treatment with different concentrations of flavonoids. CC—Cell control; Amentoflavone; Procyanidin B1; Procyanidin B2; Epicatechin; Gossypetina; Herbacetin; Procyanidin A2; Procyanidin C1; and Genistein, respectively. To determine the CC_50_, Vero cells were cultured in 96-well plates and treated with the compounds for 48 h. Cell viability was assessed using the MTT method.

Most of the tested flavonoids did not show cytotoxicity at the cytotoxic concentrations tested for 50% of the cells, even at the highest concentration (500 μM). However, Herbacetin (HBC) and Genistein (GNT) exhibited cytotoxicity at concentrations above 31.25 μM and 125 μM, respectively. The CC_50_ results provided relevant data on the bioactivity of the compounds. Higher compound concentration values indicate a lower risk of that compound being toxic to the cell. In comparison with the compounds used, we tested Amantadine as a positive control. In the cytotoxicity assay, Amantadine showed a CC_50_ of 561.26 ± 1.16 μM, showing no cytotoxicity to the cell (Figure S1). The higher this value, the greater the safety level for the use of the drug, thereby guiding subsequent experiments.

### Evaluation of Flavonoids for anti-CHIKV and anti-MAYV Activity

After evaluating the cytotoxicity of the compound on the cell and determining the working concentration that is cytotoxic to 50% of the cells (CC_50_), we performed the antiviral assay, that is, the effective protective concentration for 50% of the infected cells (EC_50_). The data on the cell viability of all flavonoids for CHIKV are shown in Figure 3.

**Figure 3:**
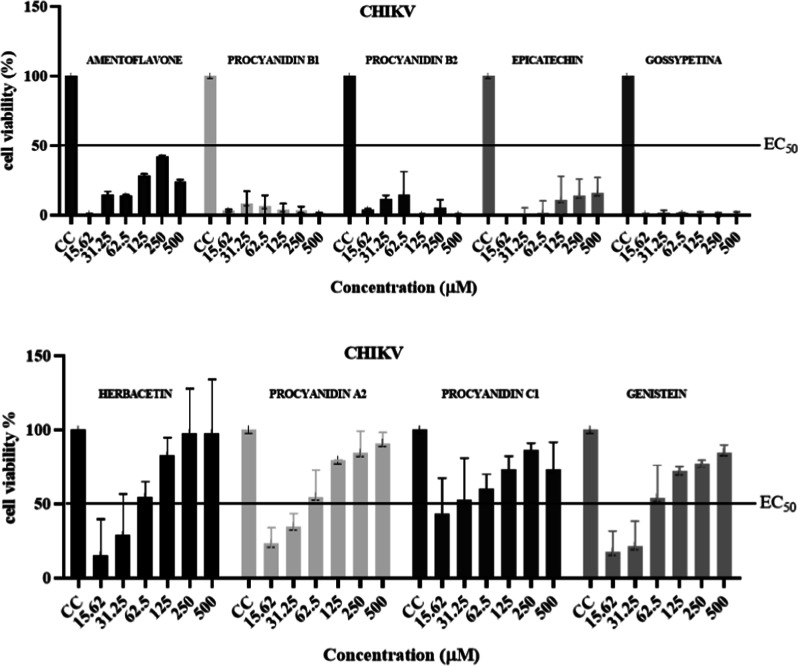
Cell viability after infection (CHIKV) and treatment with flavonoids. Vero cells were cultured in 96-well plates and after 24 h, cells were infected with the viruses (MOI: 0.1) and treated with the compounds at different concentrations for 48 h. The EC_50_ was determined after treating cells and viruses. Cell viability was assessed using the MTT method. Cells only infected were used with viral control, and 0.5% (v/v) DMSO was used as a negative control. There was no significant difference between the means of the independent assays performed by Tukey’s test at *p* > 0.05.

With the results of CC_50_ and EC_50_, we determined the selectivity index (SI). This parameter is used to determine the antiviral potential of a substance without compromising cell viability and indicates the relationship between the pharmacological and toxic effects and the effective and cytotoxic dose of the compounds under study. Amantadine was used as a positive control for CHIKV (Figure S1).

For a compound to have a notable effect on viral particles, the SI values must be greater than 1, greater than 4 to be considered optimal, and greater than 10 to be considered excellent, which is the expected and desired outcome to be considered safe and promising (Table 1).

**Table 1:** Cytotoxic Concentration (CC_50_), Effective Concentration (EC_50_), and Selectivity Index (SI) of the Flavonoids Tested Against CHIKV

flavonoids	CC_50_ (μM)	EC_50_ (μM)	SI
Amentoflavone	>500	380.6 ± 167.1	1.3
Gossypetin	498.08 ± 11.17	374.7 ± 176.3	1.3
Procyanidin B1	>500	>500	1.0
Procyanidin B2	434.72 ± 4.52	500 ± 0.10	0.9
Epicatechin	>500	>500	1.0
Procyanidin A2	337.01 ± 14.53	56.7 ± 4.80	5.9
Procyanidin C1	>500	50.2 ± 32.40	10.4
Herbacetin	58.75 ± 35.20	37.01 ± 27.80	1.6
Genistein	165.27 ± 30.73	64.4 ± 29.80	2.6
Amantadine	>500	418.98 ± 1.70	1.3

The cytotoxic concentration (CC_50_), the effective antiviral concentration (EC_50_), and the selectivity index (SI) of the flavonoids were evaluated. To determine the CC_50_, Vero cells were cultured in 96-well plates and treated with the compounds for 48 h. The EC_50_ was determined after treating cells of different concentrations and viruses (MOI: 0.1), followed by incubation for 48 h. Cell viability was assessed using the MTT method. Cells only infected were used with viral control, amantadine as positive control, and 0.5% (v/v) DMSO was used as a negative control. The SI was calculated by the ratio CC_50_/EC_50_. There was no significant difference between the independent assays (Tukey’s test at *p* > 0.05).

After primary analysis, the following four compounds that exhibited the lowest EC_50_ and best SI were selected as the most promising against CHIKV: Procyanidin (PA2), Procyanidin (PC1), Herbacetin (HBC), and Genistein (GNT).

Considering the close phylogenetic and structural relationship between CHIKV and MAYV, the four promising flavonoids were subsequently re-evaluated for their activity against CHIKV and later evaluated for their anti-MAYV activity. The results regarding MAYV are presented in Table 2. Ribavirin was used as a positive control for MAYV and showed a CC_50_ of 2142.1 ± 174.0 μM, EC_50_ of 486.4 ± 1.8 μM and SI 4.4 (Figure S2). HBC and GNT did not show high selectivity against MAYV, exhibiting higher EC_50_ values than those of the other two compounds and ribavirin. In contrast, PA2 and PC1 demonstrated higher selectivity indices, with PC1 exhibiting an EC_50_ of 8.8 μM and SI of 61.

**Table 2:** Cytotoxic Concentration (CC_50_), Effective Concentration (EC_50_), and Selectivity Index (SI) of the Flavonoids Tested against MAYV

flavonoids	CC_50_ (μM)	EC_50_ (μM)	SI
HBC	58.75 ± 35.20	257.8 ± 16.0	0.23
PA2	337.01 ± 14.53	81.7 ± 8.1	4.1
PC1	>500	8.8 ± 2.1	61
GNT	165.27 ± 30.73	263.3 ± 13.3	0.63
Ribavirin	>500	486.4 ± 1.8	4.4

The cytotoxic concentration (CC_50_), the effective antiviral concentration (EC_50_), and the selectivity index (SI) of the flavonoids were evaluated. To determine the CC_50_, Vero cells were cultured in 96-well plates and treated with the compounds for 48 h. The EC_50_ was determined after treating cells at different concentrations and viruses (MOI: 0.1), followed by incubation for 48 h. Cell viability was assessed using the MTT method. Cells only infected were used with viral control, ribavirin as positive control for MAYV and 0.5% (v/v) DMSO was used as a negative control. The SI was calculated by the ratio CC_50_/EC_50_. HBC—Herbacetin; PA2—Procyanidin A2; PC1—Procyanidin C1; and GNT—Genistein. There was no significant difference between the independent means (Tukey’s test at *p* > 0.05).

Figure 4 shows the results of infected cells viability using flavonoids against MAYV.

**Figure 4:**
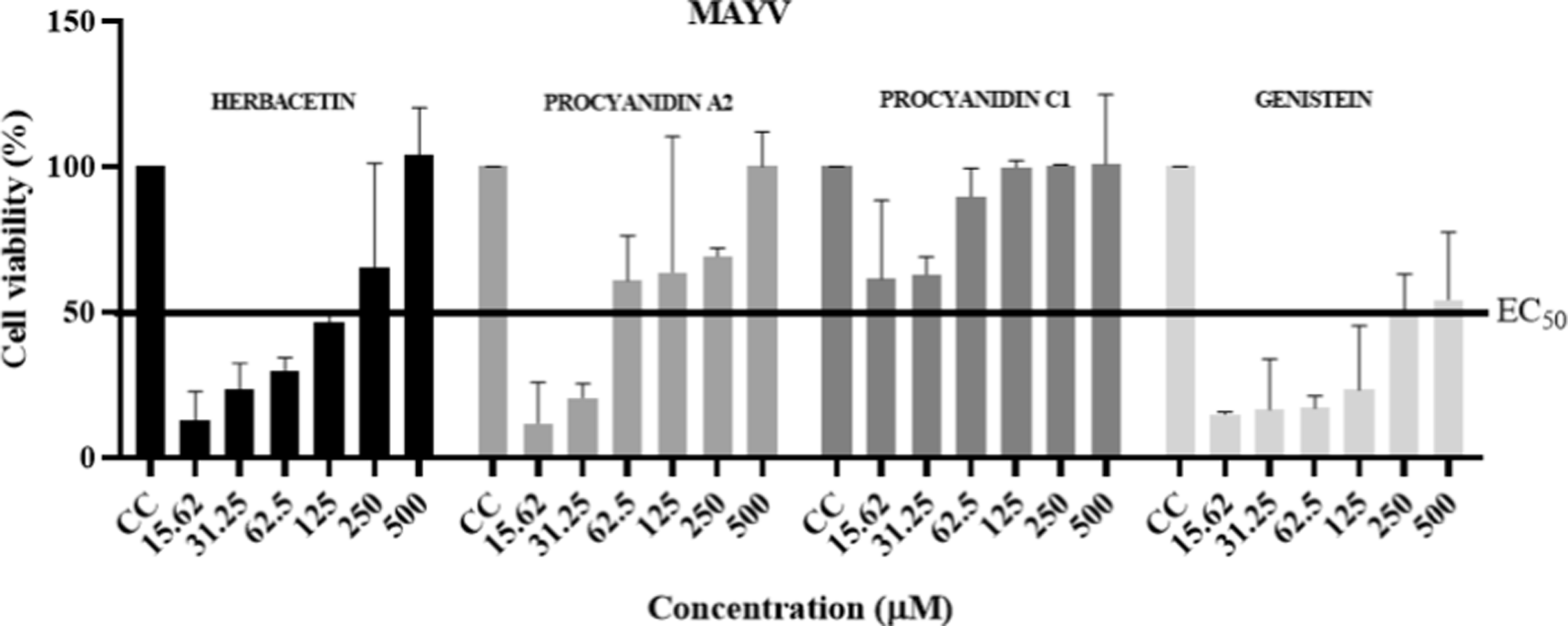
Cell viability after infection (MAYV) and treatment with flavonoids. Vero cells were cultured in 96-well plates and after 24 h, cells were infected with the viruses (MOI: 0.1) and treated with the compounds at different concentrations for 48 h. The EC_50_ was determined after treating cells and viruses. Cell viability was assessed using the MTT method. Cells only infected were used with viral control, and 0.5% (v/v) DMSO was used as a negative control. There was no significant difference between the means of the independent assays (Tukey’s test at *p* > 0.05).

In this assay, changes in cell morphology and a reduction in the viral cytopathic effect (CPE) in infected and treated cells were observed. As shown in Figure 5, the CPE was alleviated in infected cells treated with the compounds at concentration of 250 μM. Treated cells exhibited a morphology similar to that of cell control, differing from that of the viral and negative controls. Furthermore, depending on the concentration, these flavonoids demonstrated a remarkable antiviral effect against CHIKV and MAYV, as the integrity of the cell monolayer was maintained despite the infection. Cellular morphology results of the positive controls amantadine for CHIKV and ribavirin for MAYV are presented in the Figures S3 and S4, respectively.

**Figure 5:**
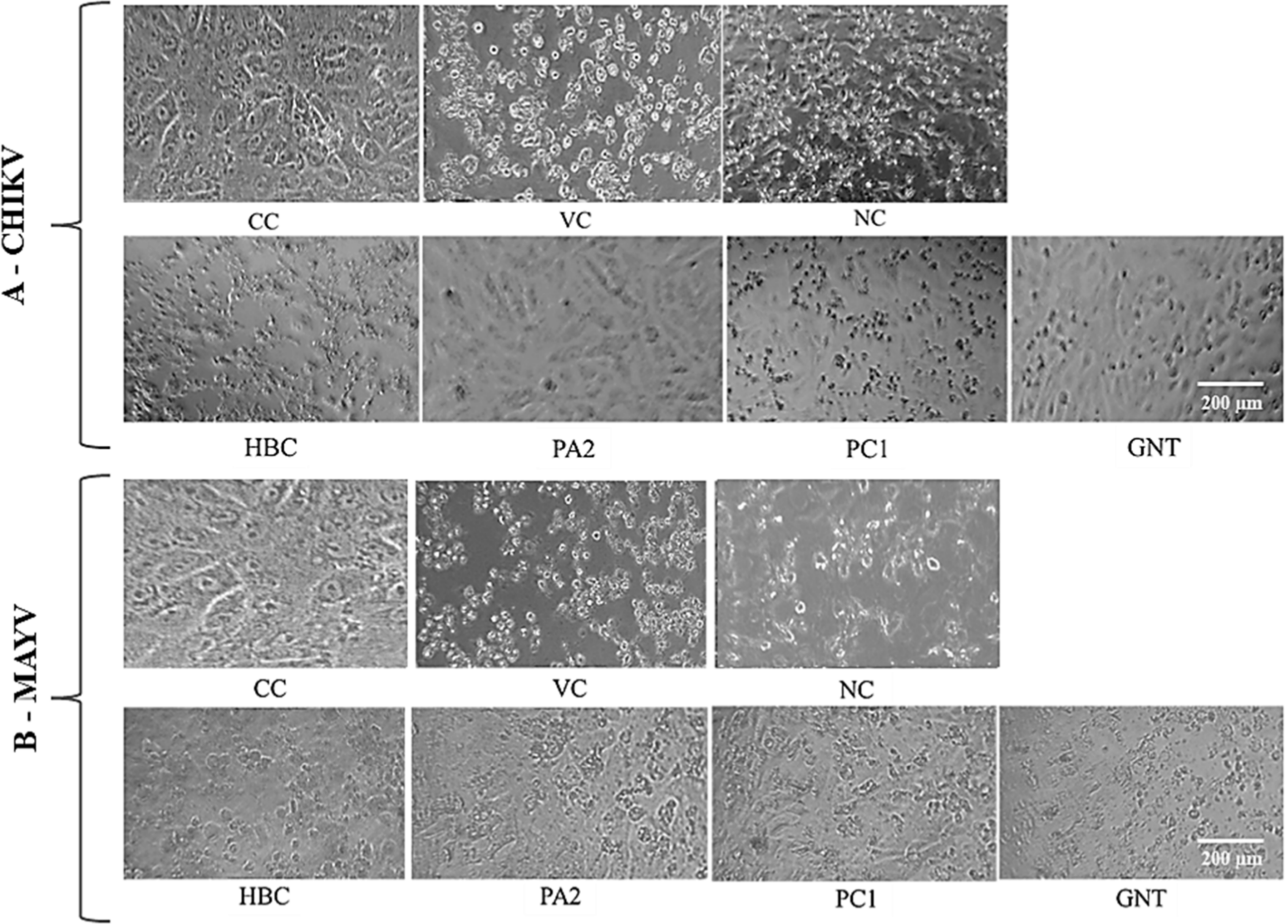
Analysis of cytopathic effect reduction by flavonoids in CHIKV (A) and MAYV (B) infected cells. Cells were infected (MOI: 0.1) and treated with the indicated flavonoids. After 48 h of infection, images were captured by optical microscopy at 200× magnification. CC—Cell control (represents uninfected and untreated cells); VC—Viral control (refers to cells infected with CHIKV or MAYV); NC—Negative control (cell with DMSO); HBC—Herbacetin 250 μM; PA2—Procyanidin A2 250 μM; PC1—Procyanidin C1 250 μM; and GNT—Genistein 250 μM. The reduction in the viral cytopathic effect indicates that flavonoids may help protect cells from destruction caused by viral infection, suggesting a possible direct antiviral effect.

These results demonstrate that the tested flavonoids could reduce virus-induced damage. These compounds not only inhibited viral replication but also provided substantial protection against infection-induced cellular stress, thereby suggesting promising therapeutic potential against both CHIKV and MAYV. Amantadine provided little protection to the cell against CHIKV. Ribavirin also protected the cells, but less effectively, with some focal areas of visible cell death (Figures S3 and S4). It is worth noting that both positive controls require a higher effective concentration to protect the cell.

### Effect of Flavonoids on Viral Yield

To evaluate the reduction in PFUs, cells were infected and treated with the compounds described in the materials and methods section. The titration results for anti-CHIKV and anti-MAYV activities are shown in Figure 6.

**Figure 6:**
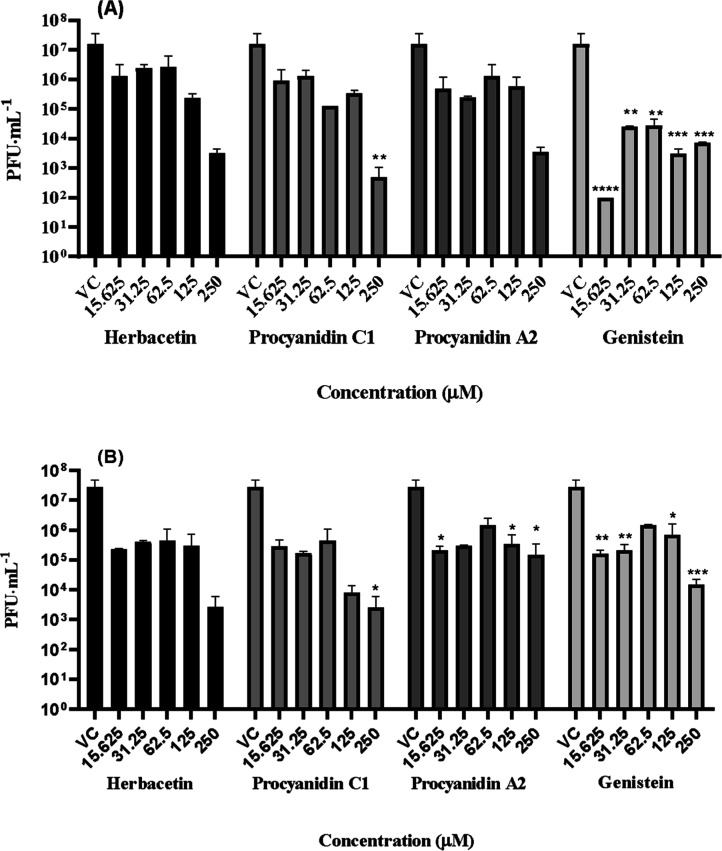
Effect of selected flavonoids on the yield of CHIKV (A) and MAYV (B) viruses. VC—untreated cells; Herbacetin; Procyanidin C1; Procyanidin A2; and Genistein, respectively. Vero cells infected with CHIKV and MAYV (MOI:0.1) were treated with different concentrations of the compounds, and the supernatants were collected after 48 h of infection for viral titration, which was performed using the Dulbecco plaque assay. The virus control (VC) consisted of infected cells that were not treated. No significant differences were observed among independent experimental replicates (Tukey’s test, *p* > 0.05). Differences relative to the viral control (VC) were analyzed by one-way ANOVA followed by Dunnett’s multiple comparisons test. *p < 0.05; **p < 0.01; ***p < 0.001; ****p < 0.0001.

The results demonstrated that all compounds reduced viral production in a concentration-dependent manner, although the magnitude and statistical significance of the effect varied according to the compound and virus evaluated. Against CHIKV (Figure 6A), Genistein (GNT) exhibited the most pronounced antiviral activity. Treatment with 15.625 μM GNT reduced viral titers by approximately 5-log units compared with the viral control, representing the largest reduction observed in this study. Significant reductions were also detected at 31.25 μM and 62.5 μM (**p < 0.01), as well as at 125 μM and 250 μM (***p < 0.001). Procyanidin C1 (PC1) significantly reduced CHIKV replication at 250 μM (**p < 0.01), resulting in an approximately 4-log reduction in viral yield. Although Procyanidin A2 (PA2) and Herbacetin (HBC) also decreased viral production, these reductions were not statistically significant when compared with the viral control (Dunnett’s test, *p* > 0.05).

For MAYV (Figure 6B), PC1 showed the strongest antiviral effect, producing an approximately 4-log reduction in viral yield at 250 μM (*p < 0.05). Significant reductions were also observed at lower concentrations, including 125 μM. PA2 significantly reduced MAYV replication at 15.625 μM, 125 μM, and 250 μM (*p < 0.05), whereas GNT showed significant antiviral activity at 15.625 μM and 31.25 μM (**p < 0.01), at 125 μM (*p < 0.05), and at 250 μM (***p < 0.001). Although HBC promoted reductions in viral titers, particularly at 250 μM, no statistically significant differences relative to the viral control were detected, however, this flavonoid was more toxic to the cell (CC_50_ = 240.62 μM). At concentrations below 250 μM, all compounds showed reductions of at least 1-log.

Overall, the anti-CHIKV and anti-MAYV activities showed similar results. At lower concentrations, the antiviral effect was reduced and varied among the compounds, with several conditions resulting in approximately 1–2 log reductions in viral. GNT and PC1 have the most notable effects against CHIKV and MAYV, respectively. These results indicate that GNT is the most effective compound against CHIKV, whereas PC1 displays superior activity against MAYV.

### Virucidal Effect

After evaluating antiviral activity and possible mechanisms of action, the study evaluated whether the action of these compounds occurred directly on the viral particles before they came into contact with the cells. The results are shown in Figure 7.

**Figure 7:**
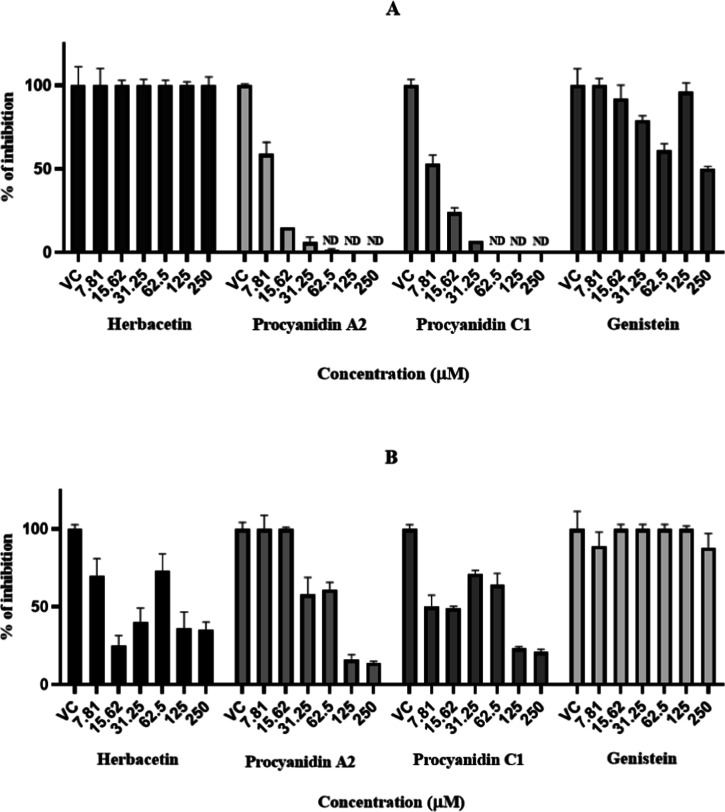
Virucidal activity of the compounds at the concentrations tested against Chikungunya (A) and Mayaro (B) viruses, respectively. VC—Viral Control; ND—not detected. The inoculum containing the virus was pretreated with flavonoids at concentrations ranging from 250 to 7.8 μM for 1 h before cell infection. Then, the cells were infected and incubated at 37 °C for 48 h. 0.5% (v/v) DMSO was used as a negative control. The percentage of inhibition (eq 1) was analyzed, considering the viral control as 100%. There was no significant difference between the means of the independent assays (Tukey’s test at *p* > 0.05).

For comparison purposes, infected cells that received no treatment (Viral Control) were established as the 100% cell lysis (total cell destruction) reference. In infected cells pretreated with the compounds (evaluation of the virucidal effect), substantial inhibition of viral plaque formation was observed for procyanidins PC1 and PA2 against CHIKV. At a concentration of 62.5 μM, no viral plaques were detected, which corresponds to 100% inhibition of the virus. Substantial reductions in viral production were maintained even at a concentration of 31.25 μM (94% for PA2 and 93% for PC1); at the concentration of 7.8 μM, which was the lowest, there was a reduction of 41% for PA2 and 47% for PC1; however, GNT showed a weaker antiviral effect (50% inhibition at 250 μM). HBC had no effect on the virus, and almost 100% of the cell lysates remained. These data indicate that these two compounds may act at different stages of infection, such as virus-cell binding or the intracellular stages of the viral cycle.

In terms of anti-MAYV activity, HBC showed pronounced activity at concentrations of 250, 125, 31.25, and 15.625 μM, with 65, 64, 60, and 75% inhibition of viral yield. Moreover, PC1 and PA2 also exhibited substantial virucidal effects at concentrations of 125–250 μM, with viral load reductions of nearly 80%. PC1 also inhibited viral production at the other concentrations tested. GNT had no significant effect on viral load reduction at any of the tested concentrations.

Altogether, PC1 was more effective in reducing infectivity against both CHIKV and MAYV. In contrast, HBC had a pronounced effect on MAYV replication but not on CHIKV replication. We believe that, although a virucidal effect was observed, some residue of the compound still exists, which may result in some antiviral activity that is not exactly virucidal. However, this is almost insignificant due to the washing step of the cell cultures in PBS buffer, after 1 h of viral adsorption, which removes most of the drug used in the treatment, thus remaining throughout the replication cycle.

### *In Silico* Assay

The four selected flavonoids were evaluated for gastrointestinal (GI) absorption, blood–brain barrier (BBB) permeability, solubility, Lipinski’s rule of five for pharmacokinetic and toxicological profiling using the SwissADME web tool. As shown in Table 3, the lipophilicity, as represented by the Log Po/w value, increased from 1.99 (HBC) to 3.47 (PC1), which may be related to greater structural hydrophobicity. Conversely, aqueous solubility (Log *S*) decreased considerably as the compounds became larger and more lipophilic, with PC1 being the least soluble (−7.39) and HBC the most soluble among the derivatives (−3.55). In terms of GI absorption, only HBC and GNT had high oral bioavailability, whereas PA2 and PC1 showed low absorption. None of the evaluated compounds demonstrated permeability across the BBB, which may be advantageous in avoiding negative effects on the central nervous system. Analysis of Lipinski’s rule of five indicated that HBC and GNT did not violate these criteria, suggesting a good drug-likeness profile for oral administration. In contrast, owing to their high molecular weights, elevated counts of hydrophilic functional groups, and low solubilities, PA2 and PC1 showed three violations. Finally, the synthetic accessibility varied among the compounds, with GNT being the most accessible (2.87) and PC1 being the most synthetically complex (6.95).

**Table 3:** Pharmacokinetic Profile of the Four Selected Flavonoids Predicted *In Silico* Using ADME-Based Computational Analysis

properties	parameters	HBC	PA2	PC1	GNT
	MW (g/mol)	302.24	576.50	866.77	270.24
	H-bond acceptors	7	12	18	5
	H-bond donors	5	9	15	3
	Molar Refractivity	78.03	144.14	219.09	73.99
Lipophilicity	Log Po/w	1.99	2.36	3.47	2.58
Water solubility	Log *S* (ESOL)	–3.55	–5.21	–7.39	–3.72
Pharmacokinetics	GI absorption	High	Low	Low	High
	BBB permeant	No	No	No	No
Drug likeness	Lipinski, Violation	Yes; 0 violations	No; 3 violations	No; 3 violations	Yes; 0 violations
Medi. Chemistry	Synthetic Accessibility	3.20	5.85	6.95	2.87

Toxicological evaluation was conducted using the ProTox protocol, focusing on hepatotoxicity, carcinogenicity, immunotoxicity, and mutagenicity (Table 4).

**Table 4:** Toxicological Profile of the Four Selected Flavonoids Predicted *In Silico* Using the ProTox Platform^a^

molecule	hepatotoxicity	carcinogenicity	immunotoxicity	mutagenicity	toxicity
HBC	Inactive (0.69)	Active (0.68)	Inactive (0.92)	Active (0.51)	Inactive (0.99)
PA2	Inactive (0.76)	Inactive (0.58)	Inactive (0.71)	Inactive (0.69)	Inactive (0,76)
PC1	Inactive (0,74)	Inactive (0.54)	Active (0.91)	Inactive (0.59)	Inactive (0.80)
GNT	Inactive (0.69)	Inactive (0.69)	Inactive (0.97)	Inactive (0.74)	Inactive (0.91)

a Values in parentheses represent probability scores, indicating the confidence level of each prediction.

Among the molecules analyzed, PA2 and GNT exhibited the most promising toxicological profiles and were classified as inactive for all evaluated categories, thereby suggesting reduced toxicological risk. The absence of hepatotoxicity, mutagenicity, or carcinogenicity signs in the *in silico* analyses further supported the potential of these molecules to advance to the preclinical stages of pharmaceutical development.

In contrast, PC1 displayed immunotoxicity, thus raising caution regarding its immunological safety. Moreover, HBC demonstrated carcinogenic activity, which is a critical risk factor and may limit its progression in the development pipeline. According to the predictive models, none of the compounds showed a high hepatotoxicity risk.

## Discussion

Considering the persistent symptoms of CHIKV and MAYV, the lack of accurate differential diagnoses, and the absence of authorized vaccines, identifying antiviral drugs to combat these two critical viruses is urgently required.^21^

In this study, the antiviral activity of nine flavonoids against CHIKV and MAYV were evaluated in Vero cells. Although the antiviral potential of flavonoids against other viruses has been well documented, studies on their antiviral effects against lesser-known viruses, such as MAYV, and their potential use as treatments for emerging arboviruses, such as CHIKV, are limited.

For a compound to have a notable effect on viral particles, the SI values must be greater than 1, greater than 4 to be considered optimal, and greater than 10 to be considered excellent, which is the expected and desired outcome to be considered safe and promising.^22^ In this study, the SI values obtained for four flavonoids (PA2, PC1, HBC, and GNT) were above this threshold, indicating that these flavonoids had low cytotoxicity and effective antiviral activity. Although potent antiviral activity is an important prerequisite for drug development, selectivity remains one of the most critical parameters for the successful translation of antiviral candidates into *in vivo* models. In antiviral drug discovery, compounds presenting Selectivity Index (SI) values below 10 are generally considered to have a narrow therapeutic window, indicating that antiviral efficacy may occur at concentrations close to those associated with cytotoxic effects. Therefore, antiviral potency alone cannot compensate for potential safety concerns. In the present study, Procyanidin C1 (PC1) emerged as the most promising candidate from a translational perspective. Against MAYV, PC1 exhibited an excellent selectivity profile (SI = 61), while against CHIKV it reached the minimum threshold generally considered favorable for further development (SI = 10.4). These findings suggest that PC1 possesses a safety margin compatible with future proof-of-concept *in vivo* studies, although additional pharmacokinetic and toxicological investigations remain necessary.

Gescher and collaborators tested the antiviral activity of *Rumex acetosa* L. extract containing oligomeric and polymeric proanthocyanidins and flavonoids against Human alphaherpesvirus 1 (HSV-1). Using antiviral activity assays in Vero cells, an SI of approximately 100 was determined, and the antiviral activity was attributed to the presence of oligomeric proanthocyanidins in the extract.^23^ In agreement with these findings, proanthocyanidin (PAC-1), a dimeric structure of methyl epigallocatechin and epicatechin, also demonstrated strong antiviral activity against MAYV, with SI values above 40.^24^ Corroborating these reports, this study revealed that procyanidin exhibited an even higher selectivity index of 61 against MAYV. In contrast, despite the remarkable antiviral activity observed for Genistein (GNT) against CHIKV, including a reduction of approximately 5 log10 in viral yield, its SI value (2.6) indicates a limited therapeutic window. Similarly, Procyanidin A2 (PA2) displayed relevant antiviral activity but only moderate selectivity (SI = 5.9 against CHIKV and SI = 4.1 against MAYV). Consequently, these compounds should not currently be considered direct candidates for *in vivo* evaluation. Instead, they may be regarded as valuable lead structures for future medicinal chemistry optimization programs aimed at increasing antiviral selectivity while reducing host-cell toxicity. However, GNT has shown antiviral potential against West Nile virus^25^ and has been investigated as a potential antiviral agent for rodent-borne arenavirus hemorrhagic fever.^26^ This supports its broad-spectrum antiviral relevance.

However, previous studies on the development of antivirals must be considered, which emphasizes that compounds with low SI values frequently fail during preclinical development due to dose-limiting toxicities, despite exhibiting strong *in vitro* antiviral activity. Therefore, improving the therapeutic window remains a critical step before advancing such molecules toward animal studies.^27–29^

Furthermore, alternative formulation approaches may contribute to overcoming these limitations. Advanced drug delivery systems, including polymeric nanoparticles, lipid nanoparticles, liposomes, and nanoencapsulation strategies, have been successfully employed to reduce systemic toxicity and improve the biodistribution of flavonoids and other natural products. Such strategies could potentially increase the therapeutic applicability of GNT and PA2 by enhancing antiviral exposure to target tissues while minimizing off-target toxicity.^30,31^ Taken together, our findings support PC1 as the most promising candidate for future *in vivo* evaluation, whereas GNT and PA2 should be considered lead compounds requiring structural optimization and/or specialized formulation strategies before further preclinical progression.

The antiviral potential of the selected flavonoids was further confirmed using a viral yield reduction assay, which confirmed their capacity to limit the production of infectious viral particles. All compounds produced a marked logarithmic reduction in viral replication, with GNT achieving a 5-log reduction at concentrations as low as 15.62 μM demonstrates a potent antiviral effect that occurs before significant cytotoxicity is reached. This suggests that while the calculated therapeutic “window” appears narrow, the functional inhibitory concentration is effective. This is a magnitude of inhibition that surpasses that of flavonoid fractions isolated from *Miconia albicans* (Sw.) Triana, which caused 1 and 2-log reductions in CHIKV load.^32^ Likewise, in the context of MAYV, PC1 displayed the most pronounced antiviral effect by inducing 5-log reductions in viral titers; however, this reduction was slightly lower than the 7-log reduction reported for the dimeric proanthocyanidin PAC1.^24^ Nonetheless, this reduction remains highly significant and reinforces the relevance of procyanidins as promising candidate structures for preclinical candidates against emerging arboviruses. Virucidal activity involves the direct inactivation of viral particles before their interaction with host cells. In this context, PC1 and PA2 showed virucidal action against CHIKV, and PC1, PA2, and HBC exhibited virucidal action against MAYV. Similarly, condensed type B procyanidin tannins derived from (−)-epicatechin were identified as virucidal compounds against severe acute respiratory syndrome coronavirus 2.^33^

To contextualize and interpret the *in vitro* results, the experimental work was complemented with *in silico*pharmacokinetic (ADME) and toxicological evaluations to appraise the drug-likeness and safety profiles of the evaluated flavonoids. Toxicological profiling revealed favorable outcomes for GNT, which was identified as a promising antiviral candidate in preclinical trials against CHIKV infection. According to the predictive data, GNT exhibited a favorable safety profile with low acute toxicity and no indications of mutagenicity, carcinogenicity, or hepatotoxicity (which classified GNT as “inactive”). These findings are consistent with previously reported clinical evidence. Randomized clinical trials investigating the use of GNT as a nutritional supplement, especially in the context of menopause and cancer, have reported a low incidence of adverse events at daily doses above 50 mg.^34,35^ In addition, a double-blind randomized study^36^ in which GNT was administered to postmenopausal women for 12 months did not detect any clinical or laboratory changes indicating systemic toxicity. Furthermore, the ADME analysis reinforced the pharmacological potential of GNT. The flavonoid exhibited good oral bioavailability, as indicated by its high predicted absorption rate, and fully satisfied Lipinski’s rule of five, which is an essential criterion for the selection of drug candidates for oral administration. Experimental data have demonstrated measurable plasma levels of GNT after oral administration, with a half-life ranging from 4–8 h in humans,^37^ which favors viable posology dosing regimens.

The predictive toxicity assessment of PC1, identified as a promising antiviral agent for MAYV, indicated a highly favorable safety profile (toxicity class 5) of low acute toxicity. These results are consistent with those in previous studies. PC1 has been tested in animal and human models as an antioxidant supplement with good tolerability. In a murine model, oral administration of PC1 at doses of up to 100 mg/kg for 30 days caused no hematological, histopathological, or behavioral alterations.^38^ In humans, extracts rich in procyanidins, including PC1, have been used as nutritional supplements, with clinical studies indicating their safety in prolonged regimens.^39–41^

The pharmacokinetic analysis of PC1 suggests that the compound can be administered orally. Although intestinal absorption is limited, its conjugated forms (glucuronides and sulfates) remain active in the body. Suitably, special formulations, such as nanoparticles or cyclodextrin complexes, can be used to improve the absorption of PC1.^42–45^

The structural diversity of the evaluated flavonoids highlights important structure–activity relationships against CHIKV and MAYV. Compounds with higher structural complexity, such as dimeric and oligomeric procyanidins (PA2 and PC1), showed greater antiviral activity and selectivity than monomeric flavonoids, suggesting that increased molecular size, degree of polymerization, and hydroxylation enhance antiviral efficacy. Notably, PC1 exhibited the highest selectivity against MAYV, reinforcing the role of oligomerization in antiviral potency. In contrast, monomeric compounds such as genistein displayed moderate activity, likely related to intracellular mechanisms rather than direct virucidal effects, but with lower selectivity. These findings are consistent with previous studies demonstrating enhanced antiviral activity of oligomeric proanthocyanidins and flavonoids with higher structural complexity,^13,23,24^ supporting their potential as scaffolds for the development of antivirals against alphaviruses.

To date, no studies have evaluated and/or cited the compounds tested in this study as antiviral agents against CHIKV and MAYV. The only exception is HBC, which was recently evaluated against CHIKV and DENV 3.^46^ The action of the tested flavonoids in this study were evaluated in comparison with that of flavonoids containing similar structures. This highlights the promising potential of flavonoids as precandidates in future preclinical analyses in the search for antivirals in the fight against arboviruses.

## Conclusion

The study findings highlight the antiviral potential of flavonoids as precandidates for preclinical trials against CHIKV and MAYV. These results identify PC1 as the most promising candidate for future preclinical development, whereas GNT and PA2 should be considered lead compounds for further optimization strategies aimed at improving selectivity and safety profiles. These compounds demonstrated favorable SIs, substantial reduction in viral replication, and potent virucidal effects.

Moreover, *in silico* analyses supported their safety, with GNT exhibiting superior pharmacokinetic properties. Collectively, these results suggest that flavonoids are valuable for developing novel antiviral strategies in preclinical trials for the development of antivirals against emerging arboviruses.

## Materials and Methods

### Cell and Viral Culture

Vero cells (African green monkey kidney epithelial cells) are widely used in arbovirus research due to their high permissiveness to viral infection. These cells lack a functional type I interferon response, allowing efficient viral replication and high viral yield, which facilitates antiviral screening and viral quantification assays.

Vero cells were obtained from the American Type Culture Collection (ATCC, Manassas, VA, USA). The cells were cultured in Dulbecco’s Modified Eagle’s Medium (DMEM) supplemented with 5% fetal bovine serum (FBS), 100 μg/mL penicillin, 100 U/mL streptomycin, and 2.5 μg/mL amphotericin B, and maintained in a humidified incubator at 37 °C with 5% CO_2_.

The African prototype strain S27 of CHIKV (1.6 × 10^7^ PFU mL^–1^) and the MAYV BeAr20290 (7.2 × 10^7^ PFU mL^–1^) strain were used to prepare the viral stocks. The viruses were propagated and titrated using the Dulbecco plaque assay^47^ and stored at −80 °C.

### Stock and Compound Preparation

The following flavonoids were used: amentoflavone (AMF), procyanidin B1 (PB1), and procyanidin B2 (PB2), which were purchased from Cayman Chemical Company (Ann Arbor, MI, USA); procyanidin A2 (PA2), procyanidin C1 (PC1), herbacetin (HBC), gossypetin (GSP), and epicatechin (EPC), which purchased from Sigma-Aldrich (St. Louis, MO, USA); and genistein (GNT), which was obtained from the Santa Cruz Biotechnology laboratory (Dallas, TX, USA). All samples were solubilized at concentrations of 5 or 10 mg/mL in a solution containing 10% dimethyl sulfoxide (DMSO), Methanol, or Chloroform, if indicated by the manufacturer. DMSO is widely used as a solvent in biological assays and is typically diluted in the culture medium so that its final concentration is significantly lower, therefore it does not exert relevant cytotoxic effects.

Amantadine hydrochloride (Momenta Farmaceutica Ltda, Brazil) was used as a positive control against CHIKV and Ribavirin was purchased from the Institute of Immunobiological Technology (Bio-Manguinhos, Brazil) and used as a positive antiviral control against MAYV.

All cells were tested and evaluated for cytotoxicity and viability. Of the nine flavonoids evaluated, the four that showed the best antiviral effects and provided the greatest cellular protection were selected for evaluation against CHIKV and MAYV.

### Cytotoxicity Assay

The 3-(4,5-dimethylthiazol-2-yl)-2,5-diphenyltetrazolium bromide (MTT) uptake assay was used to evaluate the cytotoxicity of flavonoids in Vero cells. Cells were seeded in 96-well plates at a density of 2 × 10^4^ cells/well and incubated at 37 °C with 5% CO_2_ for 24 h. The cells were treated with flavonoid concentrations ranging from 500–15.62 μM and incubated for 48 h. After incubation, the supernatant was discarded, and 25 μL of MTT was added to each well, followed by incubation for 90 min at 37 °C. Absorbance was measured at 492 nm using a Synergy HT microplate reader (Biotek Instruments, Winooski, VT, USA). Cell viability for 50% of the cells was calculated according to eq 1.

1 $$\%\;Cell\;viability=\frac{B}{A}\times 100$$

where, *A* is the absorbance of untreated cells and *B* is the absorbance of treated cells.

### Primary Evaluation and Antiviral Assay Against CHIKV and MAYV

Dose-dependent inhibition assays were performed to determine the inhibitory effect of the flavonoids and establish the half maximal effective protective concentration (EC_50_). In the first step, all nine flavonoids were tested against CHIKV. Vero cells were seeded in 96-well plates and incubated for 24 h at 37 °C with 5% CO_2_. Subsequently, the cells were treated with indicated compound concentrations and infected with CHIKV at a multiplicity of infection of 0.1 virus/cell for 48 h. Then, absorbance was measured spectrophotometrically (λ = 492 nm). The cell viability was calculated using the eq 2 below, and nonlinear regression analysis with GraphPad Prism software (version 8) was used to determine the effective protective concentration for 50% of cells (EC_50_) and Selectivity Index (SI).

2 $$\%\;Cell\;viability=\frac{\left(A-B\right)}{\left(C-B\right)}\times 100$$

where: *A* represents the OD of the treated and infected cells; *B*: untreated and infected cells; and *C*: untreated and uninfected cells.

An MOI of 0.1 was selected to reproduce a low-multiplicity infection model, which permits multiple cycles of viral replication and spread throughout the cell monolayer, thereby enhancing the detection of antiviral effects while avoiding rapid and complete cytopathic destruction of the culture.

Thereafter, the supernatants were collected, and viral plaque assays were performed to quantify the viral titers. The flavonoids that showed the best antiviral activity were selected for other assays against CHIKV and were further evaluated against MAYV by repeating the same methodology mentioned above.

The selectivity index (SI) was expressed as the ratio between the CC_50_ and EC_50_ for both virus (CHIKV and MAYV).

### Quantification of Viral Yield by Plaque Assay

Viral titers were determined using Dulbecco’s plaque assay method, with modifications.^39^ Briefly, Vero cells seeded in 24-well plates were infected with serial dilutions of supernatants collected from infected cells. The plates were incubated at 37 °C, and after 60 min of adsorption, the supernatant was discarded and 1 mL of DMEM medium with 2% FBS, antibiotics, and 1.5% (p/v) sodium carboxymethylcellulose (CMC) was added. After 48 h post-infection, viral plaque formation was assessed.

### Evaluation of the Virucidal Effect of Flavonoids

The Virucidal Effect Assay was conducted to determine the capacity of the flavonoids to directly inactivate the free viral particle (virion) before its interaction with the host cell, thereby differentiating this mechanism from intracellular antiviral effect. A viral suspension containing the CHIKV (1.6 × 10^7^ PFU.mL^–1^) or MAYV (7.2 × 10^7^ PFU.mL^–1^), at MOI 0.1, was prepared in serum-free MEM medium. The flavonoids were diluted to the same concentrations used in the standard antiviral assay (250–7.8 μM). Aliquots of the virus were mixed with each concentration of the flavonoid in separate tubes 1:1 ratio, final volume of 200 μL and incubated at 37 °C for 1 h (in a CO_2_ incubator). This incubation allows for the direct interaction of the compound with the viral surface. The negative control cells were treated with 0.5% (v/v) dimethyl sulfoxide. After this period, the pretreated viral suspensions were diluted 10x in order to reduce the presence of antiviral action and transferred to 24-well plates containing previously established Vero cell cultures. After the dilution, the virus-compound mixture was added to cell monolayers of Vero (1 × 10^5^ cell/well) in 24-well plates. The inoculum volume was 100 μL. The plates were incubated for 1 h at 37 °C for viral adsorption. After 1 h, the cell monolayer was washed with PBS 1X to remove the residual and the remaining unadsorbed viruses. Then an overlay of semisolid culture medium was added. At 48 h, viral lysis plaque formation was assessed as previously described to determine the vírus yields. The reduction in viral titer relative to negative control (virus + vehicle) was used to calculate the percentage of virucidal inhibition. The percentage inhibition (% *I*) was calculated using eq 3

3 $$\%\;I=\left[1-\left(\frac{A}{CV}\right)\right]\times 100$$

where *A* is the number of viral plaques observed in infected and treated cells, and CV is the number of plaques in infected and untreated cells.

### Data Analysis

Analysis of variance (ANOVA) was used to analyze differences among group means, followed by Dunnett’s test for comparisons of treated samples with untreated samples and Tukey test was performed for post hoc analysis to differentiate among pairs groups. Statistical significance was fixed at *p* < 0.05. The mean absorbance values were used for nonlinear regression analysis in cytotoxicity and antiviral assays. All statistical analyses performed using Experimental triplicates were performed in two independent assays, with the results from the independent assays shown as mean and standard deviation. OriginPro 10 software (OriginLab Corporation, Northampton, MA, USA) and GraphPad Prism 8 (GraphPad Software Inc., San Diego, CA, USA) were used for data analysis.

### *In Silico* Assay

To determine the viability of the study compounds for pharmaceutical development, molecular structures were initially obtained in a Simplified Molecular Input Line Entry System format using the MolView platform (https://app.molview.com/). Subsequently, predictive analyses were performed using SwissADME software^40^ and ProTox-3.0 ^41^ to estimate parameters related to safety, absorption, distribution, metabolism, and excretion (ADME) and potential toxicity, respectively.

## Supplementary Material



## Notes

Additional experimental details, materials, and methods, including figures of experimental results (PDF)
