# Supplementary material for: Evaluation of the Antiviral Activity of Monomeric, Dimeric, and Oligomeric Flavonoids against Chikungunya and Mayaro Viruses
Source: ACS Omega. 2026 Jul 16;11(29):43669–81. doi: 10.1021/acsomega.6c02683 (PMC13425342; doi:10.1021/acsomega.6c02683)
Supplement: Supplementary file 1 [file ao6c02683_si_001.pdf]

**Evaluation of the Antiviral Activity of Monomeric, Dimeric, and Oligomeric Flavonoids Against Chikungunya and Mayaro Viruses**

Delaine Meireles Gouvêa[a]; Maria Cecília Muniz de Souza Brand [a]; Millena Alves Máximo Vaz [a]; Priscila Gonçalves Ferreira [a]; Vinicius Viana [a]; Kamilly Soares de Almeida [a]; Ariane Coelho Ferraz [b]; Adriana Cotta Cardoso Reis [c]; Geraldo Célio Brandão [c]; Cintia Lopes de Brito Magalhães [b]; José Carlos de Magalhães [a*]

[a] Universidade Federal de São João del-Rei Campus Alto Paraopeba, Departamento de Química, Biotecnologia e Engenharia de Bioprocessos, Rod. MG 443, KM 7 Fazenda do Cadete, Ouro Branco - MG, CEP: 36495-000, Ouro Branco, Minas Gerais, Brazil.
[b] Universidade Federal de Ouro Preto, Programa de Pós-Graduação em Ciências Biológicas, Núcleo de Pesquisas em Ciências Biológicas, R. Diogo de Vasconcelos 122, Pilar, Ouro Preto - MG, CEP: 35400-000, Brazil.
[c] Universidade Federal de Ouro Preto, Programa de Pós-Graduação em Ciências Farmacêuticas, Escola de Farmácia, R. Diogo de Vasconcelos 122, Pilar, Ouro Preto - MG, CEP: 35400-000, Brazil.

*E-mail: josecarlos@ufsj.edu.br

## Supporting Information

Additional experimental details, materials, and methods, including figures of experimental results.

## Material and Methods

### Reference drug for CHIKV and MAYV

Amantadine hydrochloride 100 mg (Momenta Farmaceutica Ltda, São Paulo, Brazil). A 100 mg sample of amantadine hydrochloride was initially dissolved in 1 mL of ultrapure water and subsequently diluted in DMEM-HG medium to obtain a final stock concentration of 25 mg.mL$^{-1}$. The resulting solution was employed in both the cytotoxicity and in vitro antiviral assays against CHIKV, which were evaluated using the MTT colorimetric method.

Ribavirin was purchased from the Institute of Immunobiological Technology (Bio-Manguinhos, Brazil) and used as a positive antiviral control against MAYV.

## Results

### Cytotoxicity assay CHIKV

$CC_{50}$ = 84.89 ± 1.16 µg.mL$^{-1}$ (561.26 ± 1.16 µM)

### *In vitro* antiviral assay CHIKV

$EC_{50}$ = 63.37 ± 1.70 µg.mL$^{-1}$ (418. 98 ± 1.70 µM)

IS = 1.34

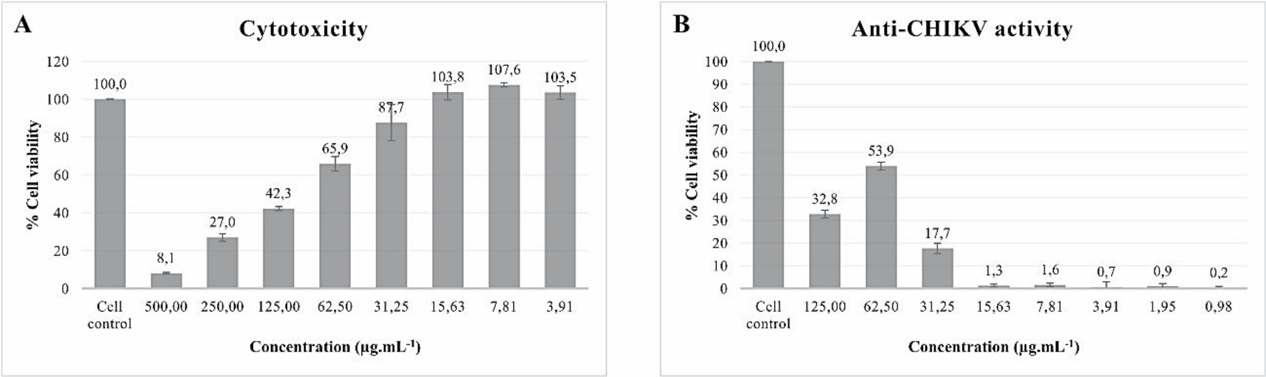


**Figure S1** – Cytotoxicity (A) and anti-CHIKV activity (B) of the amantadine determined using MTT method in Vero cells, with the respective 50% cytotoxic concentration ($CC_{50}$) values (µg.mL$^{-1}$).

**Cytotoxicity assay MAYV**

$CC_{50}$ = 523.1 ± 42.49 µg.mL$^{-1}$ (2142.1 ± 174 µM)

***In vitro* antiviral assay MAYV**

$EC_{50}$ = 118.8 ± 1.98 µg.mL$^{-1}$ (486.4 ± 8.1 µM)

IS = 4.43

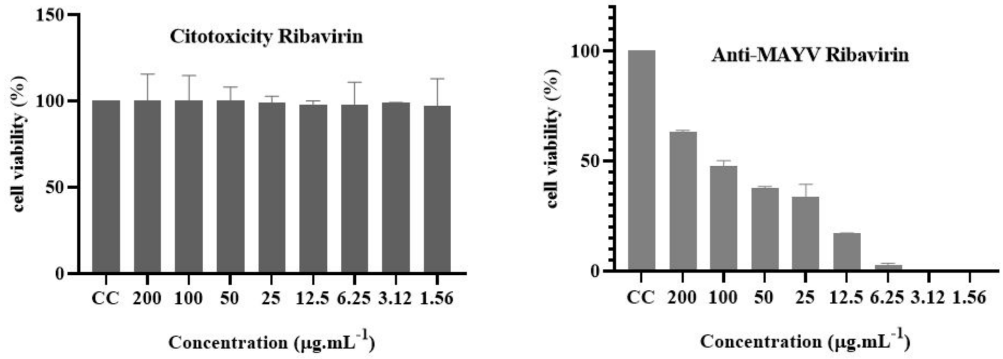


**Figure S2 –** Cytotoxicity and anti-MAYV activity of the ribavirin determined using the MTT method in Vero cells, with the respective 50% cytotoxic concentration ($CC_{50}$) values (µg.mL$^{-1}$).

**Inhibitory cytopathic effect anti-CHIKV and anti-MAYV**

The inhibitory cytopathic effect assay was performed in Vero cells using amantadine at a concentration of 70 μg.mL$^{-1}$ (462.81 μM), which is close to the $EC_{50}$ value previously determined in the antiviral activity assay against CHIKV.

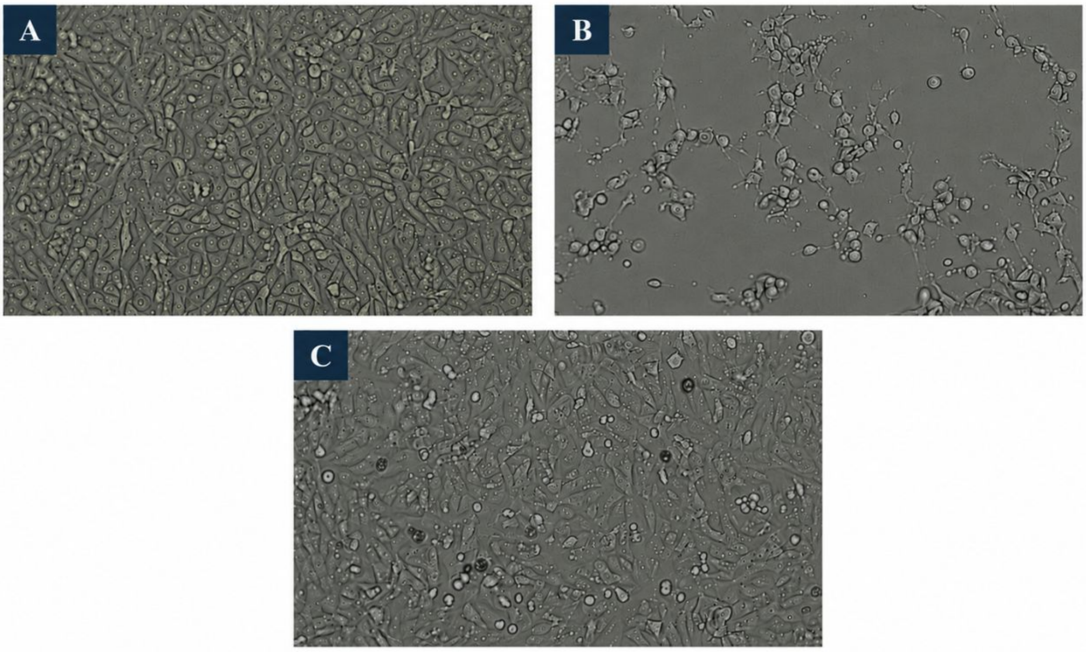


**Figure S3 –** Inhibitory cytopathic effect of the positive control amantadine against CHIKV in Vero cells, 48 hours post-infection. A: Cell Control, B: Viral Control, C: Antiviral activity ($EC_{50}$) of amantadine against CHIKV (70 μg.mL$^{-1}$). Magnification, 100x.

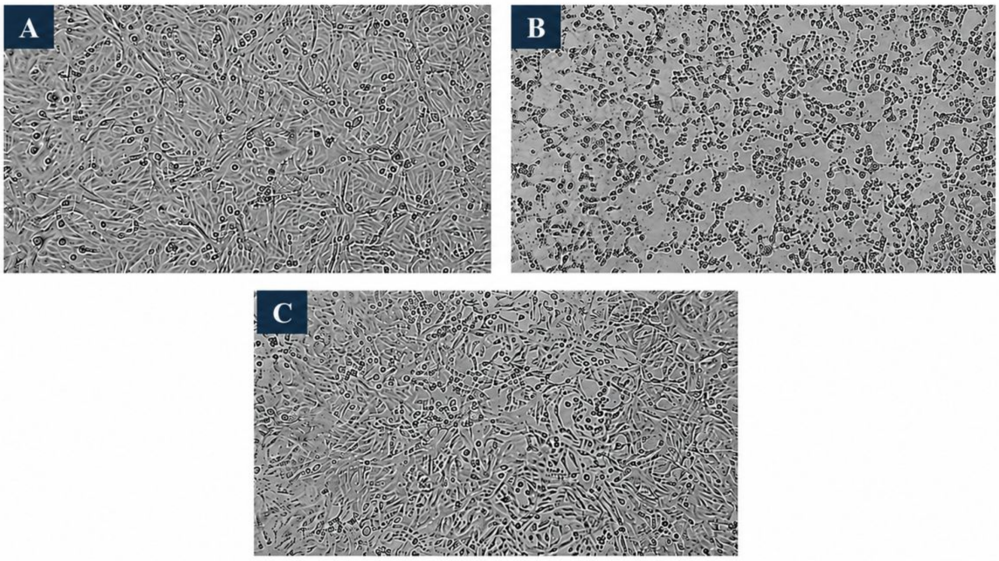


**Figure S4 –** Inhibitory cytopathic effect of the positive control ribavirin against MAYV in Vero cells, 48 hours post-infection. A: Cell Control, B: Viral Control, C: Antiviral
